# Novel Insight into the Serum Sphingolipid Fingerprint Characterizing Longevity

**DOI:** 10.3390/ijms23052428

**Published:** 2022-02-22

**Authors:** Pietro Barbacini, Enrica Torretta, Beatrice Arosio, Evelyn Ferri, Daniele Capitanio, Manuela Moriggi, Cecilia Gelfi

**Affiliations:** 1 Department of Biomedical Sciences for Health, University of Milan, 20133 Milan, Italy; pietro.barbacini@unimi.it (P.B.); daniele.capitanio@unimi.it (D.C.); 2 IRCCS Istituto Ortopedico Galeazzi, 20161 Milan, Italy; enrica.torretta@grupposandonato.it; 3 Department of Clinical Sciences and Community Health, University of Milan, Via Pace 9, 20122 Milan, Italy; beatrice.arosio@unimi.it; 4 Geriatric Unit, Fondazione IRCCS Ca’ Granda Ospedale Maggiore Policlinico, Via Pace 9, 20122 Milan, Italy; evelyn.ferri@guest.unimi.it; 5 Gastroenterology and Digestive Endoscopy Unit, IRCCS Policlinico San Donato, San Donato Milanese, 20097 Milan, Italy; manuela.moriggi@grupposandonato.it

**Keywords:** sphingolipids, mass spectrometry, nitric oxide, ROS, longevity, aging, centenarians

## Abstract

Sphingolipids (SLs) are structural components of the lipid bilayer regulating cell functions. In biological fluids, their distribution is sex-specific and is at variance in aging and many disorders. The aim of this study is to identify SL species associated with the decelerated aging of centenarians. SLs, extracted from serum of adults (Ad, 35–37 years old), aged (Ag, 75–77 years old) and centenarian (C, 105–107 years old) women were analyzed by LC-MS/MS in combination with mRNA levels in peripheral blood mononuclear cells (PBMCs) of SL biosynthetic enzymes. Results indicated in Ag and C vs. Ad a comparable ceramides (Cers) increase, whereas dihydroceramide (dhCer) decreased in C vs. Ad. Hexosylceramides (HexCer) species, specifically HexCer 16:0, 22:0 and 24:1 acyl chains, increased in C vs. Ag representing a specific trait of C. Sphingosine (Sph), dihydrosphingosine (dhSph), sphingosine-1-phosphate (S1P) and dihydrosphingosine-1-phosphate (dhS1P), increased both in Ag and C vs. Ad, with higher levels in Ag, indicating a SL fine-tuning associated with a reduced physiological decline in C. mRNA levels of enzymes involved in ceramide de novo biosynthesis increased in Ag whereas enzymes involved in sphingomyelin (SM) degradation increased in C. Collectively, results suggest that Ag produce Cers by de novo synthesis whereas C activate a protective mechanism degrading SMs to Cers converting it into glycosphingolipids.

## 1. Introduction

The serum sphingolipidome is characterized by hundreds of lipid species with different origins and molecular properties. As they are structural components of the lipid bilayer, sphingolipids are bioactive molecules involved in signal transduction regulating apoptosis, autophagy, differentiation, senescence, and inflammatory responses [1,2,3]. Ceramide is the central hub, acting as a second messenger in cellular signaling pathways with beneficial or detrimental effects for cell survival [4,5,6]. In biological fluids, abundance of SLs is sex-specific and characterizes dementia, cardiovascular diseases (CVD), diabetes (T2DM), obesity, susceptibility to viral infections and osteoporosis [7,8,9,10,11,12]. In physiology, the comparative assessment of SL levels made it possible to reveal changes in aging, identifying molecules potentially able to predict a better trajectory of aging or evolution toward CVD, T2DM [13,14] or disabilities as osteoarthritis [15].

In the last two decades centenarians increased significantly, particularly women that are up to 83.5% of the total centenarians’ population in Europe [16]. Their morbidity is restricted in a short time frame compared to aged subjects [17], representing an exceptional model for the study of longevity. However, the reason centenarians remain functionally independent and in good health for nearly 96% of their life [18] is not entirely understood. It is known that inflammaging in long-lived people is lower compared to aged subjects enabling them to counteract the physiological decline facilitating their recovery from stressor events [19,20,21]. Lipidomic profiles have been addressed in many studies to identify species responsible for the decelerated aging of centenarians. However, results were at variance [22,23,24], due to enrollment of different cohorts and to preanalytical and analytical approaches that influence the quali/quantitative assessment of SLs in biological fluids. From literature, a consensus regarding the sphingolipidome signature of successful aging is missing, and authors tend to agree that sex differences profoundly impact circulating SLs, making gender a confounding factor that can influence results [10,25,26].

Molecular investigations at the genetic, epigenetic, metabolomic and immunologic levels addressed the role of reactive oxygen species (ROS) to unravel putative molecular characteristics of centenarians’ advantage [27,28,29]. In this context, centenarians appeared to be less susceptible to ROS accumulation compared to aged subjects [30,31].

Recently, we demonstrated that levels of thioredoxin reductase 1 (TRXR1), controlling oxide and peroxide production, were comparable in centenarians and 70-year-old women [32]. At variance, levels of mitochondrial protein nitrosylation and alcohol dehydrogenase 5 (ADH5/GSNOR), controlling nitrosative stress and senescence, were comparable in centenarians and young subjects [32], suggesting that ADH5/GSNOR could be directly involved in successful aging [33,34,35]. However, the relationship between stress response and lipid profile associated with a long life is still not addressed. Ceramide-activated phosphatases have been shown to act on vascular endothelial growth factor signaling, hindering the activation of endothelial nitric oxide synthase [36]. This suggests a possible role of some SL bioactive species in the protection from nitrosative stress.

This study aims to characterize the serum sphingolipid profile in aged compared to long-lived subjects and discuss the possible relationship between SLs and nitrosative stress. Knowing the sex specificity of SL distribution in blood [37], we analyzed SLs in serum from the same groups of women investigated at the protein level in a previous study (adults, aged and centenarians) [32] by untargeted and targeted LC-MS/MS. mRNA levels of a set of enzymes involved in the SL biosynthetic pathway were assessed in circulating peripheral blood mononuclear cells (PBMCs) to identify preferential pathways involved in successful aging, achieving better insight into the molecular longevity signature.

## 2. Results

### 2.1. Participants

The investigated subjects were all normal weight women grouped as adult (Ad, 35–37 years old), aged (Ag, 75–77 years old) and centenarians (C, 105–107 years old) enrolled in our previous study [32]. Concerning medications, 12 Ag subjects (80%) and 11 C subjects (73.3%) had hypertension and were under antihypertensive treatment. Among those, 7 Ag (46.6%) and 7 C subjects (46.6%) were also under cardio-protector treatment. Anthropometric characteristics and medications of the three groups are shown in Table 1, while specific characteristics of single participants are reported in Supplementary Table S1.

### 2.2. Glyco/Sphingolipid LC-MS/MS Analysis

**Figure 1:**
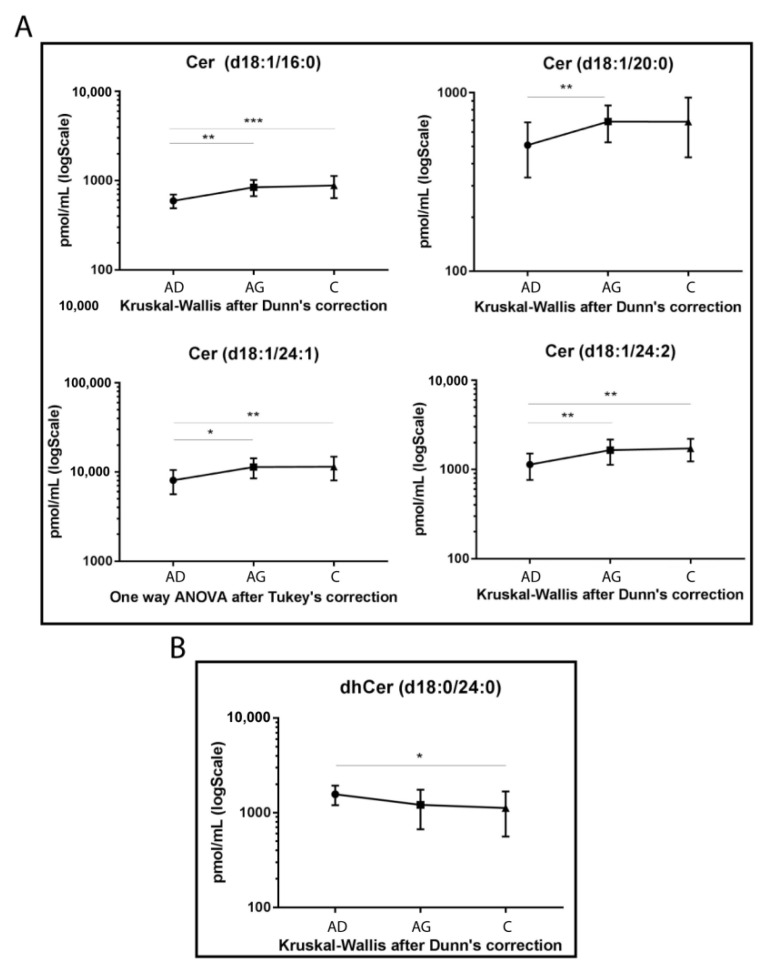
Untargeted MS analysis results for Cers (**A**) and dhCer d18:0/24:0 (**B**). Results are expressed as pmol/mL in Log scale (Ad n. 15, Ag n.15, C n.15). *p*-values are indicated as: * *p*-value < 0.05, ** *p*-value < 0.01 and *** *p*-value < 0.001.

The LC-MS/MS analysis was conducted on sphingolipid extracted from 15 Ad, 15 Ag and 15 C serum samples in triplicate, following mild alkaline hydrolysis method to eliminate phospholipids and reduce the dynamic range [38]. The analysis identified a total of 39 sphingolipid species. Results from ceramide levels are shown in Figure 1A. Cer 16:0 increased in both Ag and C compared to Ad (*p-*value < 0.01 and <0.001 respectively). The same trend was observed for Cer 24:1 (*p-*values < 0.01 in C vs. Ad and <0.05 in Ag vs. Ad) and for Cer 24:2 (*p-*values < 0.01, for both C vs. Ad and Ag vs. Ad). Moreover, Cer 20:0 showed the same trend but was statistically significant in Ad vs. Ag (*p-*value < 0.01), only.

Conversely, dhCer (d18:0/24:0) was the only species that reached a statistically significant variation, with a decreasing trend in C vs. Ad (*p-*value < 0.05) (Figure 1B).

**Figure 2:**
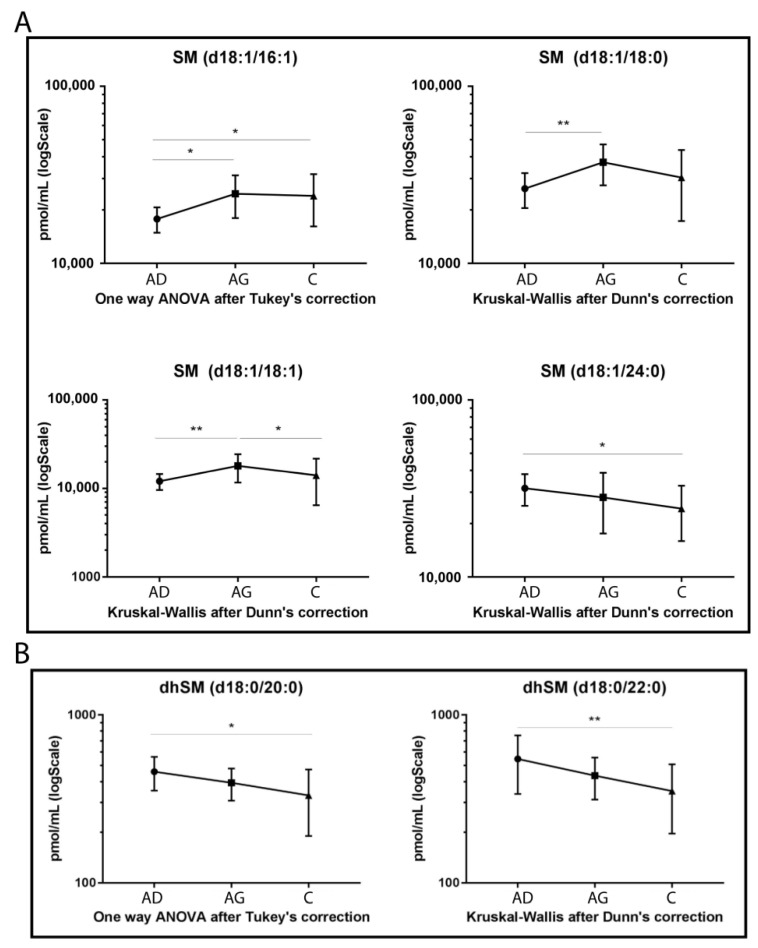
Untargeted MS analysis results for SMs (**A**) and for dihydrosphingomyelins (dhSMs) (**B**). Results are expressed as pmol/mL in Log scale (Ad n. 15, Ag n.15, C n.15). *p*-values are indicated as: * *p*-value < 0.05 and ** *p*-value < 0.01.

Regarding SMs (Figure 2A), four species showed statistically significant variation among groups. SM d18:1/16:1, SM d18:1/18:0 and its unsaturated counterpart, SM d18:1/18:1, increased in Ag compared to Ad (*p-*value < 0.05, 0.01 and 0.01 respectively). In C vs. Ag, SM d18:1/18:1 decreased (*p-*value < 0.05), whereas SM 16:1 increased in C vs. Ad (*p-*value < 0.05). Regarding SMs long chains, SM d18:1/24:0 showed a decreasing trend in aging, becoming significant in C vs. Ad (*p-*value < 0.01), only.

Figure 2B shows that both dhSMs acyl chains d18:0/20:0 and d18:0/22:0 decreased with a similar trend in C vs. Ad (*p-*value < 0.05 and *p-*value < 0.01, respectively). 

**Figure 3:**
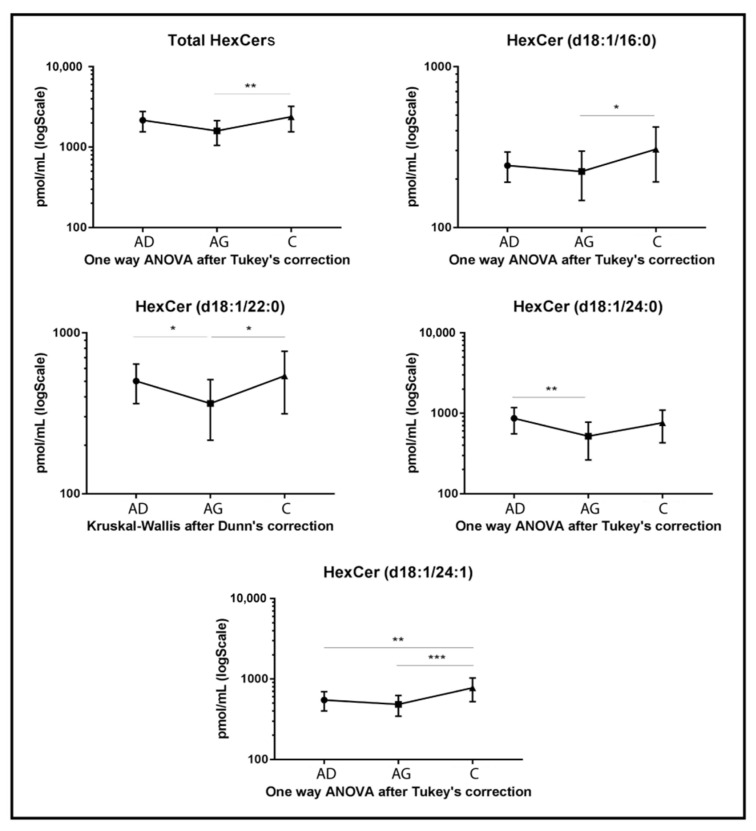
Untargeted MS analysis results for HexCers. Results are expressed as pmol/mL in Log scale (Ad n. 15, Ag n.15, C n.15). Total value is expressed as the sum of all quantified acyl chains of the selected sphingolipid. *p*-values are indicated as: * *p*-value < 0.05, ** *p*-value < 0.01 and *** *p*-value < 0.001.

Regarding glycosphingolipids, HexCers, dihexosylceramides (dihexCers) and monosialoganglioside GM3 (GM3) variations were observed among groups. Specifically, total levels of HexCers (Figure 3) and several acyl chains had a peculiar trend. Total HexCers and HexCer’s acyl chains d18:1/16:0, d18:1/22:0 and d18:1/24:1 increased in C compared to Ag (*p-*value < 0.01, *p-*value < 0.05, *p-*value < 0.05 and *p-*value < 0.001, respectively). HexCer 24:1 also increased in C compared to Ad (*p-*value < 0.01), while HexCer 22:0 and HexCer acyl chain 24:0 decreased in Ag compared to Ad (*p-*value < 0.05 and 0.01 respectively).

**Figure 4:**
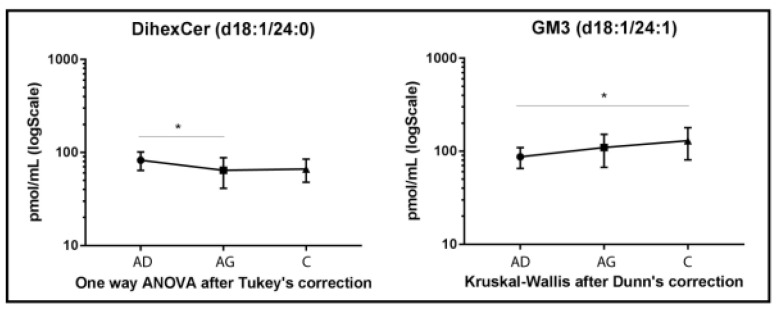
Untargeted MS analysis results for diHexCer d18:1/24:0 and GM3 d18:1/24:1. Results are expressed as pmol/mL in Log scale (Ad n. 15, Ag n.15, C n.15). *p*-values is indicated as: * *p*-value < 0.05.

DiHexCer results are shown in Figure 4. A lower variation compared to other glyco/sphingolipid classes was observed, with decreased levels of diHexCer 18:1/24:0, in Ag vs. Ad (*p-*value < 0.05). A different trend characterized GM3 acyl chain d18:1/24:1, which increased in C vs. Ad (*p-*value < 0.05) only (Figure 4).

**Figure 5:**
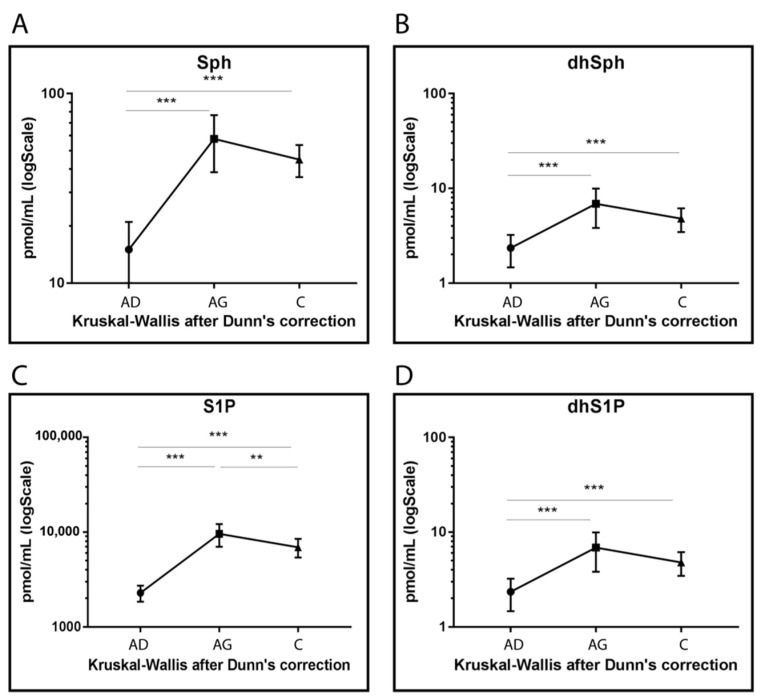
Targeted MS analysis results for Sph (**A**), dhSph (**B**), S1P (**C**) and dhS1P (**D**). Results are expressed as pmol/mL in Log scale (Ad n. 15, Ag n.15, C n.15). *p*-values are indicated as: ** *p*-value < 0.01 and *** *p*-value < 0.001.

Sph, DhSph, S1P and dhS1P levels increased in Ag and C compared to Ad (*p* < 0.001) even though lower levels were observed for S1P in C compared to Ag (*p-*value < 0.001) (Figure 5).

### 2.3. Glyco/Sphingolipid Biosynthetic Pathway in PBMCs

To assess in Ag and C changes of enzymes controlling the glyco/sphingolipid biosynthetic pathway, mRNA expression was quantitatively assessed in peripheral blood mononuclear cells. 

**Figure 6:**
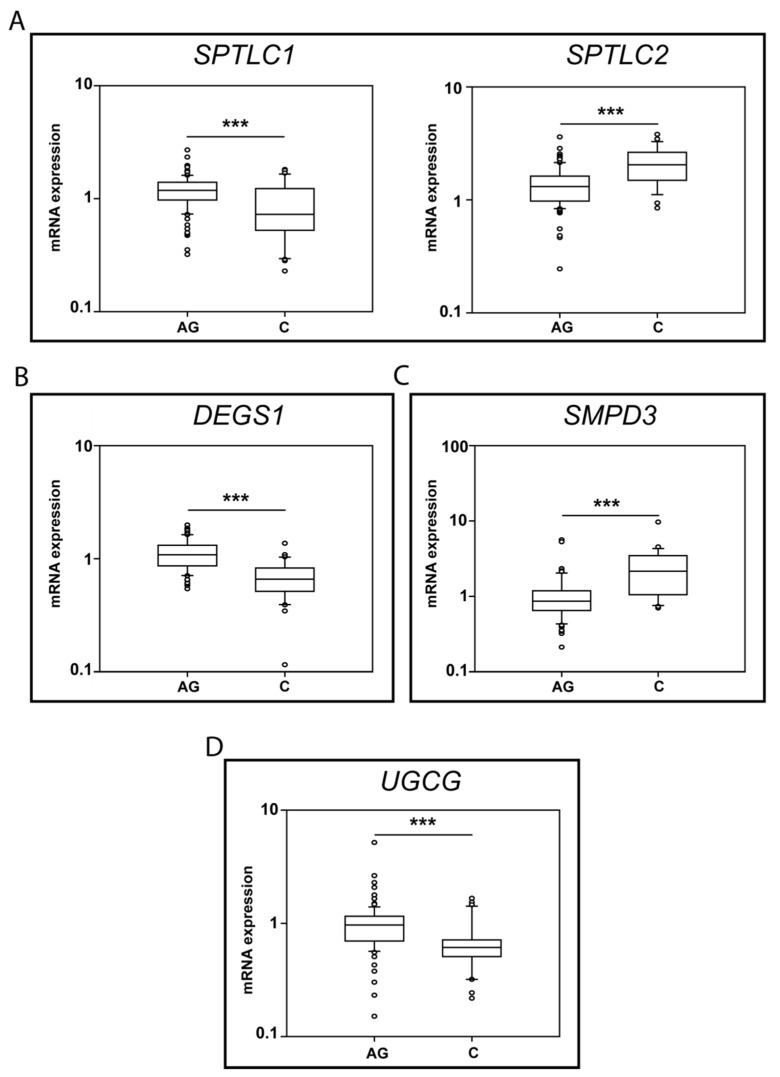
mRNA levels for *SPTLC1* and *2* (**A**), *DEGS1* (**B**), *SMPD3* (**C**) and *UGCG* (**D**) (Ag n.91, C n. 30). *p*-values is indicated as: *** *p*-value < 0.001.

Significant changes in Ag vs. C were observed for: *SPTLC1*, *SPTLC2*, *DEGS1*, *SMPD3* and *UGCG*, and box-plot results are shown in Figure 6.

Regarding the initial step of de novo sphingolipid synthesis, *SPTLC1* mRNA levels decreased in C (*p-*value < 0.001) compared to Ag, whereas *SPTLC 2* increased in C vs. Ag (*p-*value < 0.001) (Figure 6A).

mRNA levels of *DEGS1*, the limiting enzyme controlling the conversion of dhCer to Cer, decreased in C compared to Ag (*p-*value < 0.001) (Figure 6B). Conversely, neutral sphingomyelinase II (*SMPD3*) mRNA levels increased in C vs. Ag (*p-*value < 0.01) (Figure 6C), whereas other enzymes involved in SMs degradation, neutral SMase III (*SMPD4*) and the acidic SMase (*SMPD1*), were unchanged between C and Ag (Supplementary Figure S1).

Concerning glucosyl transferase (*UGCG*), mRNA levels decreased in C compared to Ag (*p-*value < 0.05) (Figure 6D).

### 2.4. Immunoblotting of *UGCG*

**Figure 7:**
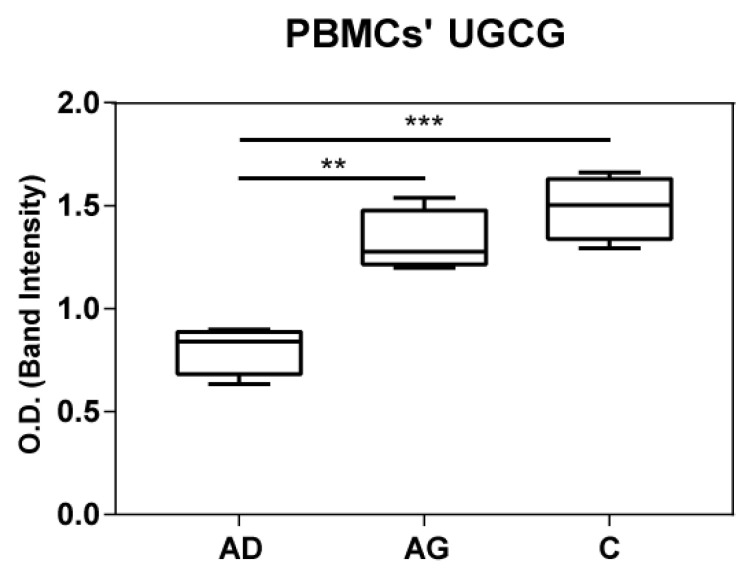
UGCG protein expression in PBMC from immunoblotting. *p*-values are indicated as: ** *p*-value < 0.01 and *** *p*-value < 0.001.

To shed light on the apparent discrepancy between *UGCG* mRNA levels in PBMCs and HexCer levels in serum, UGCG was assessed at protein level in both PBMCs and serum by immunoblotting. Results indicated increased levels of UGGC in both C and Ag compared to Ad (Figure 7 and Supplementary Figure S2). In serum UGGC was undetectable as shown in Supplementary Figure S3.

## 3. Discussion

Aging is a condition involving the entire organism in which cells undergo a physiological decline irrespectively to their origin. Circulating sphingolipids belong to membranes of extracellular vesicles, including exosomes and large plasma membrane-derived microvesicles [39] also released from PBMCs in the bloodstream. We hypothesized that the serum sphingolipidome, mirroring the whole organism status, will provide a signature of the physiological decline and of better aging.

Hypertension and pharmacological treatments (anti-hypertensives and cardio-protective drugs) characterize Ag and C compared to Ad. It is well known that sphingolipids, as well as many other lipid classes, are influenced by treatments [40,41] and atherosclerosis [42,43]. However, hypertension is a common condition in people >60 years old. According to the National Health and Nutrition Examination Survey in 2020, more than 74% of the older population had hypertension [44] and required blood pressure reduction and cardio-protector treatment to decrease the risk of CVD and death [45,46,47,48,49]. Regarding hypertension prevalence and CVD risk management in our cohort 80% of Ag subjects and 73.3% of C subjects were under treatment, according to the prevalence of these disorders in the general older population, thus they represented a realistic sample group.

Results indicated that increase of Cers was comparable in Ag and C vs. Ad subjects. Cers levels, specifically Cer C16, C24:1 and C24:2, increased both in Ag and C compared to Ad, whereas dhCer decreased in C compared to Ad. It should be of note that the increment of Cers in C is not proportional in Ag compared to Ad. It is noteworthy that C are 30 years older than Ag and a further Cers increase will be expected. In this context, dhCer decrease could be associated with the capacity of C to maintain Cer’s homeostasis [50]. Furthermore, glucosylceramide species, specifically HexCer 16:0, HexCer 22:0 and HexCer 24:1 chains, increased in C compared to Ag and specifically characterized C vs. both Ag and Ad. A recent paper highlighted the relevance of HexCer as a protective molecule to maintain cellular integrity in colon epithelial cells in response to stressors [51], and HexCers increase was described in cerebrospinal fluid (CSF) from idiopathic normal pressure hydrocephalus patients, compared to Alzheimer’s diseases subjects [9]. It is tempting to speculate that this increase could be a beneficial aspect at large, protecting C from stressor events. 

Aged women were also characterized by higher level of SM acyl chain 18:1/18:1 compared to C. Sph, dhSph, S1P and dhS1P, increased both in Ag and C vs. Ad, with higher levels in Ag. These results suggest a fine-tuning of sphingolipid levels in C that could be associated with their ability to maintain a proper balance between stressor events and protective mechanisms. An extensive study of the lipidomic profile in men and women in aging [37] indicated a strong association with the increase of ceramide and deoxy-ceramide species, particularly in women. The authors suggested an atypical de novo synthesis of ceramide, which involves alanine instead of L-serine as a precursor amino acid in the first step of palmitoyl-CoA conjugation. In agreement with our results, the study described a modest positive association between aging and levels of HexCers, GM3s and SMs in women.

Preliminary results from MRM of Cers and HexCers in PBMCs’ extracts, (Supplementary Figure S4) showed similar results of Cers and HexCers species in PBMCs and serum of Ad, Ag and C supporting the study of the enzymatic pathway regulating sphingolipid synthesis and degradation by means of mRNA transcripts.

The origin of peculiar differences and similarities in sphingolipids abundance in Ag and C in serum, was addressed by analyzing in PBMC mRNA levels of enzymes involved in the sphingolipid metabolic/catabolic pathway. Aged subjects were characterized by increased levels of *SPTLC1* and *DEGS1*, both enzymes promote de novo Cer synthesis and ceramide accumulation, confirming serum results. Ceramide is responsible for insulin resistance and steatosis [52], therefore it is tempting to speculate that accumulation of ceramide in elderly unbalances their sphingolipid profile towards a less favorable picture. Centenarians, on the other hand, were characterized by increased levels of *SPTLC2*, also involved in ceramide de novo biosynthesis. It has been demonstrated that the adenoviral expression of Sptlc2 increases ceramide levels in vitro, activating the stress-activated c-Jun N-terminal kinase (JNK) inhibiting insulin signal. However, the same paper indicated that *SPTLC2* increased insulin sensitivity in vivo [53], suggesting that a subtle protective mechanism could be activated in C to maintain glucose homeostasis through the activation of *SPTLC2*. Further investigations are ongoing to clarify this issue.

Furthermore, enzymes responsible for SMs degradation characterize C. Neutral sphingomyelinase II (*SMPD3*) increased in C compared to Ag, suggesting that Cer levels are not mainly produced by de novo synthesis but result from the conversion of SM to Cer. This hypothesis is supported by a decreased trend of SMs levels in C compared to Ag and by lower levels of SM d18:1/18:1. Conversely, mRNA levels of neutral sphingomyelinase 3 (*SMPD4*) and aSMase (*SMPD1*) were unchanged in Ag vs. C (Supplementary Figure S1). However, increased levels of aSMase in aged subjects compared to Ad were observed in a previous study from our group [54], supporting the hypothesis that aSMase increased in aging and was not further increased in C. From these results, it can be postulated that increased levels of *SMPD3* are specific of C.

*UCGC* mRNA levels decrease in C compared to Ag, in contrast with increased levels of HexCer in C. To clarify our findings, immunoblotting of UGCG in PBMC extracts from Ad, Ag and C were performed. Results indicated an increase of UGCG in Ag and C compared to Ad. Comparable protein levels were observed in Ag and C, although a slight decrease in Ag was seen. High protein levels of UGCG in C suggest that HexCer levels could exert a feedback inhibition on *UCGC* transcription. Additionally in this case, further studies at the cellular level are in progress to clarify this issue. 

By analyzing results from this study, we can postulate that Ag actively produce Cers by de novo pathway according to *SPTLC1* and *DEGS1* increase. In contrast, C degrade SM to Cers, which in turn can be transported from the endoplasmic reticulum (ER) to the Golgi, converted into glycosphingolipids (HexCer) by UGCG and subsequently metabolized to highly complex glycosphingolipids such as gangliosides [55]. 

Another aspect that we would like to discuss is the relation of SL composition and nitrosative/oxidative dysregulation detected in sera of the same subjects (aged and centenarian women) in our previous study [32]. Reports of a relation between sphingolipids and nitric oxide (NO) were addressed in the 1990s, in which ceramides were associated with decreased NO production in alveolar macrophages [56] and GM3 induction was associated with NO release in peritoneal macrophages [57]. The interplay between nitric oxide and ceramide accumulation was assessed in a previous study from our group on glioblastoma multiforme cell lines. The study demonstrated that NO exposure results in a different ceramide distribution and protein expression, providing a rationale for a possible crosstalk between SLs and NO [58]. The gatekeeper of RNS is the ADH5/GSNOR system that maintains the mitochondrial protein nitrosylation and mitochondrial fragmentation under control counteracting mitophagy and senescence [33]. Serum protein nitrosylation has been recently assessed in the same group of Ad, Ag and C women [32] and a lower level of mitochondrial protein nitrosylation was observed in C compared to Ag. In addition, C were characterized by levels of ADH5/GSNOR comparable to Ad subjects, indicating that C can counteract nitrosative stress and promote longevity [32,33]. Furthermore, it has been demonstrated that NO is a potent inhibitor of nSMase [59], suggesting that high NO levels can prevent SM degradation through nSMase. C are characterized by higher levels of ADH5/GSNOR and lower levels of protein nitrosylation compared to Ag, suggesting lower levels of endogenous NO supporting the hypothesis that nSMase activity is further enhanced in C promoting the conversion of Cers to HexCers [32,60,61]. An extensive study on cellular models is ongoing.

On the other hand, aging is not only characterized by RNS increase but also by reactive oxygen species (ROS), and, in this context, it is of note that the thioredoxin TRXR1 oxidoreductase system, controlling the thiol redox homeostasis, acts as an antioxidant system through removal of H_2_O_2_ by peroxiredoxins (Prx) [62]. In our previous study, TRXR1 decreased equally in C and Ag, suggesting that a redox unbalance is present at the same level in aged and centenarians [32], supporting the idea that physiologically, high levels of ROS characterize aging at large [63,64]. It has been also demonstrated that ROS stimulates nSMase activity [65], further supporting the increase of neutral sphingomyelinase in centenarians. 

Complex gangliosides increased in C and Ag subjects. The GM3 progressive increment in aged and centenarians must account for ceramide metabolism, which includes, besides ceramide synthase, also ganglioside GD3 resulting in the formation of gangliosides. This species promotes ROS generation and apoptosis, also supported by aSMase activation [66]. Ganglioside synthesis is not controlled by the ADH5/GSNOR system. However, it is influenced by unbalance of the TRXR1 redox system promoting ROS generation, a process that is still active in centenarians as in aged subjects. More detailed studies are required to precisely address this issue, particularly comparing data from other cohorts in which the unbalance of the redox system is present. 

**Figure 8:**
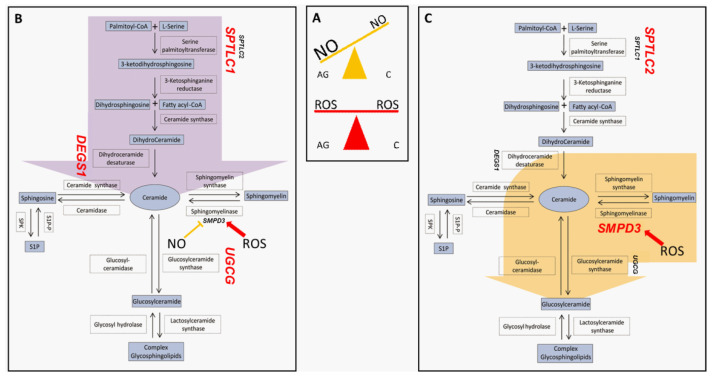
Nitro/oxidative state in aged and centenarians (box (**A**)) and proposed preferential pathways determining sphingolipids fate in aged subjects (**B**) and centenarians (**C**). Light blue boxes indicate lipids; white boxes indicate enzymes involved in the pathway, while unboxed acronyms indicate gene names. Gene names are highlighted in red when their mRNA expression in PBMC is higher in the AG vs. C comparison (box (**B**)) or in the C vs. AG comparison (box (**C**)). Aged subjects seem to be prone to increase ceramide levels through de novo biosynthesis (purple arrow), while centenarians convert sphingomyelin-derived ceramide into complex glyco/sphingolipids (yellow arrow).

To summarize, the peculiarity of centenarians is that they can cope very efficiently with nitrosative stress, as demonstrated by our previous study, keeping under control levels of NO, mitochondrial protein nitrosylation and activating HexCer synthesis, avoiding the production of Cer by de novo synthesis (Figure 8).

This is the first study that addresses in the same group of long-lived subjects the sphingolipid profiles and their biosynthetic pathway. The study also discusses the possible relationship between SLs and NO in centenarians. However, the study did not consider the possibility for some aged subjects to become centenarians, and in this context, a longitudinal study would have been meaningful. Unfortunately, the sampling from enrolled subjects did not include their follow-up. Nevertheless, it is known that the loss of study participants (attrition) is a common, well-known challenge in longitudinal studies [67], resulting in a 77% (36% to death, 21% to drop-out, 20% to sickness) cohort loss during a ten years follow-up as showed by Jacobsen et al. [68]. The major drawback is represented by the absence of mRNA data on PBMCs of adult subjects; however, the focus of this study was the definition of a different sphingolipid profile between Ag and C. We are also aware of the limitation regarding the restricted number of subjects; unfortunately, long-healthy aging is an extraordinary condition and recruitment of subjects is a difficult task. Despite novelties proposed by our study, further detailed studies in the field of successful aging and longevity are in progress to define more precisely the role of complex glyco/sphingolipids and their relationship with nitrosative stress.

## 4. Materials and Methods

### 4.1. Subjects Recruitment and Ethical Statement

Serum samples collected from 45 female subjects were grouped according to subjects’ age at the time of sampling into adults (35–37; *n* = 15, median age: 37 years old, AD), aged (75–77; *n* = 15, median age: 78 years old, AG) and centenarians (105–107; *n* = 15, median age: 105.8 years old, C). All enrolled subjects gave their informed consent for inclusion in the study. The study was approved by the Ethics Committee of the Fondazione Istituto di Ricovero e Cura a Carattere Scientifico (IRCCS) Ca’ Granda Ospedale Maggiore Policlinico, Milan (Protocol identification code No. 2035, amendment 30/11/2011) and of the IRCCS Fondazione Don Carlo Gnocchi, Milan (Project identification code No. 2017-0622, amendment 03/04/2018), and all procedures were conducted in accordance with the Declaration of Helsinki.

### 4.2. Reagents and Chemicals

UPLC-MS Methanol and UPLC-MS grade water were from Thermo fisher scientific (Waltham, MA, USA), while chloroform, 3,5-Di-tert-4-butylhydroxytoluene (BHT) and ammonium formate were purchased from Sigma-Aldrich (Saint Louis, MO, USA). Potassium hydroxide was from Merk Millipore (Burlington, MA, USA). Acetic and formic acid were from Fluka Analytical (Honeywell, Morris Plains, NJ, USA).

### 4.3. Sphingolipid Extraction

One hundred µL of serum for each sample were mixed with 0.1 mL of ultrapure water and 1.5 mL of a 0.01% (*w*/*v*) Butylated hydroxytoluene (BHT), methanol/chloroform 2:1 solution, fortified with internal standards (200 ppm of sphingomyelin (d18:1/12:0), ceramide (d18:1/12:0), sphingosine (d17:1), sphingosine-1-phosphate (d17:1) and glucosyl (β)ceramide (d18:1/12:0) from AVANTI polar lipids (Avanti Polar Lipids, Alabaster, AL, USA) and extracted overnight at 48 °C under shaking. After extraction, 0.15 mL of potassium hydroxide 1M were added and samples were incubated at 37 °C for two hours. Solutions were then neutralized with 0.15 mL of acetic acid 1M and dried under a nitrogen stream. Sphingolipids were resuspended in methanol, transferred to a clean tube and dried using a speedvac. A total of 0.15 mL of methanol were added and after brief centrifugation at 10,000 *g* for 3 min, supernatants were stored in glass vials at −80 °C.

### 4.4. Untargeted LC-MS for Sphingolipids Analysis

Ten μL of Sphingolipid extracts were injected, separated and analyzed using a Waters Aquity UPLC system coupled to a Waters Synapth G2-Si (Waters, Millford, MA, USA) operating in positive electrospray ionization mode. Full scans were obtained in a 50 to 1500 Da windows. Accuracy and reproducibility were maintained employing an independent reference spray via LockSpray. A C8 Acquity UPLC BEH (Waters) 100 mm × 2.1 mm id, 1.7 µm column was used to separate sphingolipid extracts following a gradient: 0.0 min: 80% B, 3 min: 90% B, 6 min: 90% B, 15 min: 99% B, 18 min: 99% B, 20 min: 80% B, at 0.3 mL/min flow rate. Phases were composed as follows: (A) 2 mM ammonium formate in water, 0.05 mM formic acid; (B) 1 mM ammonium formate in methanol 0.05%mM formic acid. Compounds were identified based on mass accuracy with an error < 5 ppm, the retention time compared to that of a standard (±2%), and MS/MS spectra of common fragments. Mass spectra were analyzed by MassLynx™ 4.2 Software (Waters), and lipids were annotated as lipid subclasses as follows (sphingosine backbone/number of carbon atoms of the fatty acid: amount of unsaturation of the fatty acid. MS/MS spectra were acquired and assigned as species based on precursor ions and product ions m/z 264.268 and m/z 266.286, corresponding to sphingosine backbone (d18:1) and dihydrosphingosine backbone (d18:0), respectively.

### 4.5. Multiple Reaction Monitoring LC-MS (MRM-MS)

Sphingosine, dihydrosphingosine, S1P and dihydroS1P were quantified using a Xevo TQ-S micro mass spectrometer (Waters). Extracts were injected and separated on a C8 Acquity UPLC BEH 100 mm × 2.1 mm id, 1.7 µm (Waters) hold at 30 °C, using a gradient: 0.0 min—80% B; 3 min—90% B; 6 min—90% B; 15 min—99% B; 18 min—99% B; 20 min—80% B, at 0.3 mL/min flow. Phase A and phase B were the same as for untargeted LC-MS analysis. An electrospray interface operating in positive ion mode was employed to obtain MS/MS spectra by acquiring MRM transitions spectra of: sphingosine d17:1, 286.40 > 250.40, sphingosine d18:1, 300.40 > 264.40, sphingosine d18:0 302.4 > 266.4, cone voltage 40 V, collision energy 16 eV; sphingosine-1-phosphate d17:1, 366.40 > 250.40, sphingosine-1-phosphate d18:1, 380.40 > 264.40, sphingosine-1-phosphate d18:0 382.4 > 266.4, cone voltage 20 V, collision energy 16 eV. Capillary voltage was set at 3.5 kV, while source temperature was 150 °C. The desolvation gas flow was set to 1000, and the desolvation temperature was set to 350 °C. Data acquisition and data analysis were performed with MassLynx™ 4.2 (Waters).

### 4.6. Sphingolipids Gene Expression

PBMCs were isolated by density gradient (Lympholyte-H, Cedarlane, Burlington, Ontario, Canada). mRNA levels were assessed in two different batches; the first, representing 15 AG and 15 C subjects, was employed to assess difference in levels of enzymes involved in sphingolipids biosynthesis. The second batch, consisting of a larger cohort (91 AG and 30 C subjects), was employed to confirm and further expand previous results. Total RNA was extracted from PBMCs using Chomczynski and Sacchi’s modified method. Two micrograms of total RNA was reverse-transcribed using the SuperScript VILOTM cDNA Synthesis Kit (Invitrogen by Thermo Fisher Scientific). For Serine Palmitoyltransferase Long Chain Base Subunit 1 (*SPTLC1*), Serine Palmitoyltransferase Long Chain Base Subunit 2 (*SPTLC2*), Serine Palmitoyltransferase Long Chain Base Subunit 3 (*SPTLC3*), Sphingomyelin Phosphodiesterase 1/acid sphingomyelinase (*SMPD1*), Sphingomyelin Phosphodiesterase 3/neutral sphingomyelinase (*SMPD3*), Sphingomyelin Phosphodiesterase 4 (*SMPD4*), Sphingomyelin Synthase 1 (*SGMS1*), Ceramide Synthase 2 (*CERS2*), Sphingosine Kinase 1 (*SPHK1*), UDP-Glucose Ceramide Glucosyltransferase (*UGCG*), Delta 4-Desaturase, Sphingolipid 1 (*DEGS1*) and ST3 Beta-Galactoside Alpha-2,3-Sialyltransferase 5 (*ST3GAL5*) gene expression analysis, quantitative PCRs were performed in the OpenArray^®^ system QuantStudio 12K Flex Real-Time PCR System (Applied Biosystems by Thermo Fisher Scientific). The list of commercial probes employed to quantify SLs’ mRNA expression levels is presented in Supplementary Table S2. Three genes have been selected as endogenous according to their stable expression in human cells (*GAPDH*, *ACTB* and *18S*) and included into the OpenArray^®^ chip. A total of 120 ng of every cDNA sample (1.2 μL of each) was mixed with 1.3 μL of PCR-grade water and 2.5 μL of TaqMan™ OpenArray^®^ Real-Time PCR Master Mix (Applied Biosystems by Thermo Fisher Scientific). Samples were loaded in duplicate into OpenArray^®^ plates. For gene expression analysis, Ct values were obtained using the Thermo Fisher ConnectTM (Thermo Fisher Scientific) online application and the Relative Quantification (RQ) software.

### 4.7. Immunoblotting

Sera from Ad, Ag and C were albumin-depleted according to the manufacturer’s instructions using the Pierce Albumin Depletion Kit (Thermo Fisher Scientific). 

Cells-pellet corresponding to 2.5 × 10^5^ cells for each subject were lysed according to the manufacturer’s instructions using the Pierce™ Mass Spec Sample Prep Kit for Cultured Cells (Thermo Fisher Scientific). Serum and PBMCs’ protein extract concentration were quantified by BCA protein assay (Thermo Fisher Scientific), and, for each group, samples were randomly selected and pooled into 1 pool (6 subjects each). Pooled samples were mixed 1:1 with 2xX loading buffer (125 mM Tris, 4% SDS, 10% glycerol and 200 mM DTT) and boiled for 5 min at 95 °C.

Protein extracts (50 µg) were loaded in quadruplicate and resolved on 12% polyacrylamide gels. Blots were incubated with rabbit primary antibody anti-Glucosylceramide synthase (BIOSS Antibodies, Woburn, MA, USA 1:1000). After washing, membranes were incubated with anti-rabbit (1:10,000. GE Healthcare, Chicago, IL, USA secondary antibodies conjugated with horseradish peroxidase. Signals were visualized by chemiluminescence using the ECL Prime Detection Kit and the Image Quant LAS 4000 (GE Healthcare) analysis system. Band quantification was performed using the Image Quant TL v. 8.1(GE Healthcare) software followed by statistical analysis (ANOVA + Tukey, *p-*value < 0.05). Band intensities were normalized against the total amount of proteins stained by Sypro ruby total-protein stain.

## Figures and Tables

**Table 1:** Anthropometric characteristics and pharmacological treatments of study participants. Data are described by median and interquartile range (if continuous) or counts (if categorical).

	Adult	Aged	Centenarians
N.	15	15	15
Age	37 (35.5–37.5)	78 (77.5–80)	105 (105–106.5)
Gender	F	F	F
Body mass index (BMI)	21.4 (19.3–22.1)	21.7 (20.2–22.9)	21.17 (20.2–23.8)
Hypertension	0/15	12/15	11/15
Anti-hypertensives	0/15	12/15	11/15
Cardioprotectors	0/15	7/15	7/15

## Supplementary Materials

The Supplementary Materials are available online at https://www.mdpi.com/article/10.3390/ijms23052428/s1.

## Abbreviations

SLs	Sphingolipids
AD	Adults
AG	Aged
C	Centenarians
Cer	Ceramide
dhCer	Dihydroceramide
HexCer	Hexosylceramide
Sph	Sphingosine
DhSph	Dihydrosphingosine
S1P	Sphingosine-1-phosphate
dhS1P	Dihydrosphingosine-1-phosphate
SM	Sphingomyelin
CVD	Cardiovascular diseases
T2DM	Type 2 diabetes
ROS	Reactive oxygen species
TRXR1	Thioredoxin reductase 1
ADH5/GSNOR	Alcohol Dehydrogenase 5
GlcCer	Glucosylceramide
PBMC	Peripheral blood mononuclear cell
BMI	Body Mass Index
dhSM	Dihydrosphingomyelin
DihexCer	Dihexosylceramide
GM3	Monosialoganglioside GM3
SPTLC1	Serine Palmitoyltransferase Long Chain Base Subunit 1
SPTLC2	Serine Palmitoyltransferase Long Chain Base Subunit 2
SPTLC3	Serine Palmitoyltransferase Long Chain Base Subunit 3
SMPD1	Sphingomyelin Phosphodiesterase 1/acid sphingomyelinase
SMPD3	Sphingomyelin Phosphodiesterase 3/neutral sphingomyelinase 2
SMPD4	Sphingomyelin Phosphodiesterase 4/neutral sphingomyelinase 3
SGMS1	Sphingomyelin Synthase 1
CERS2	Ceramide Synthase 2
SPHK1	Sphingosine Kinase 1
UGCG	UDP-Glucose Ceramide Glucosyltransferase
DEGS1	Delta 4-Desaturase Sphingolipid 1
ST3GAL5	ST3 Beta-Galactoside Alpha-2,3-Sialyltransferase
CSF	Cerebrospinal fluid
JNK	Stress-activated c-Jun N-terminal kinase
ER	Endoplasmic reticulum
NO	Nitric oxide
RNS	Reactive nitrogen species
O--	Superoxide anion radical
H_2_O_2_	Hydrogen peroxide
PTEN	Phosphatase and tensin homologue
PRX	Peroxiredoxins
