# Supplementary figures and images for: Novel Insight into the Serum Sphingolipid Fingerprint Characterizing Longevity
Source: Int J Mol Sci. 2022 Feb 22;23(5):2428. doi: 10.3390/ijms23052428 (PMC8910653; doi:10.3390/ijms23052428)

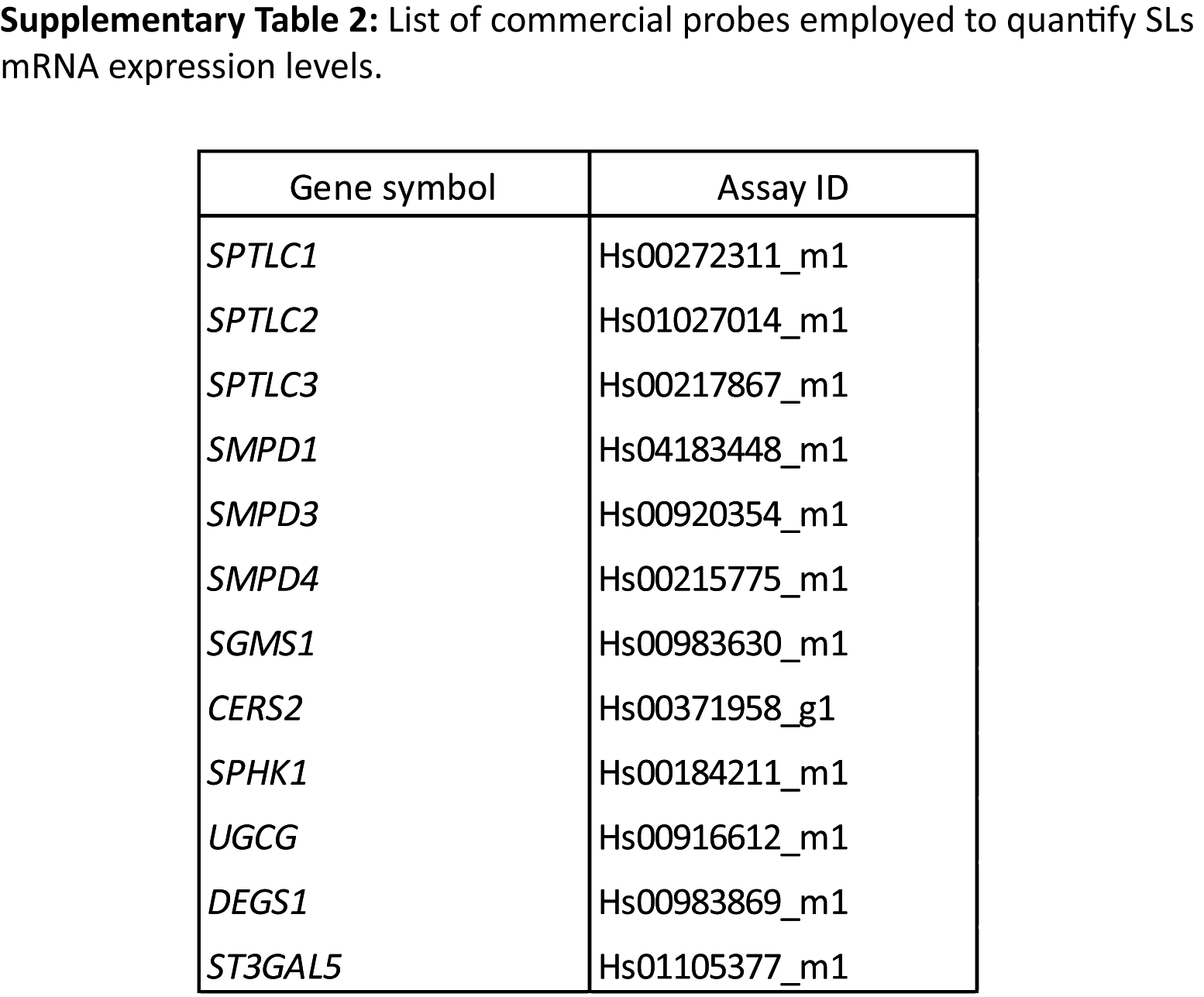

Supplement: Supplementary file 1 [file ijms-23-02428-s001.zip › Supplementary Table 2.tif]

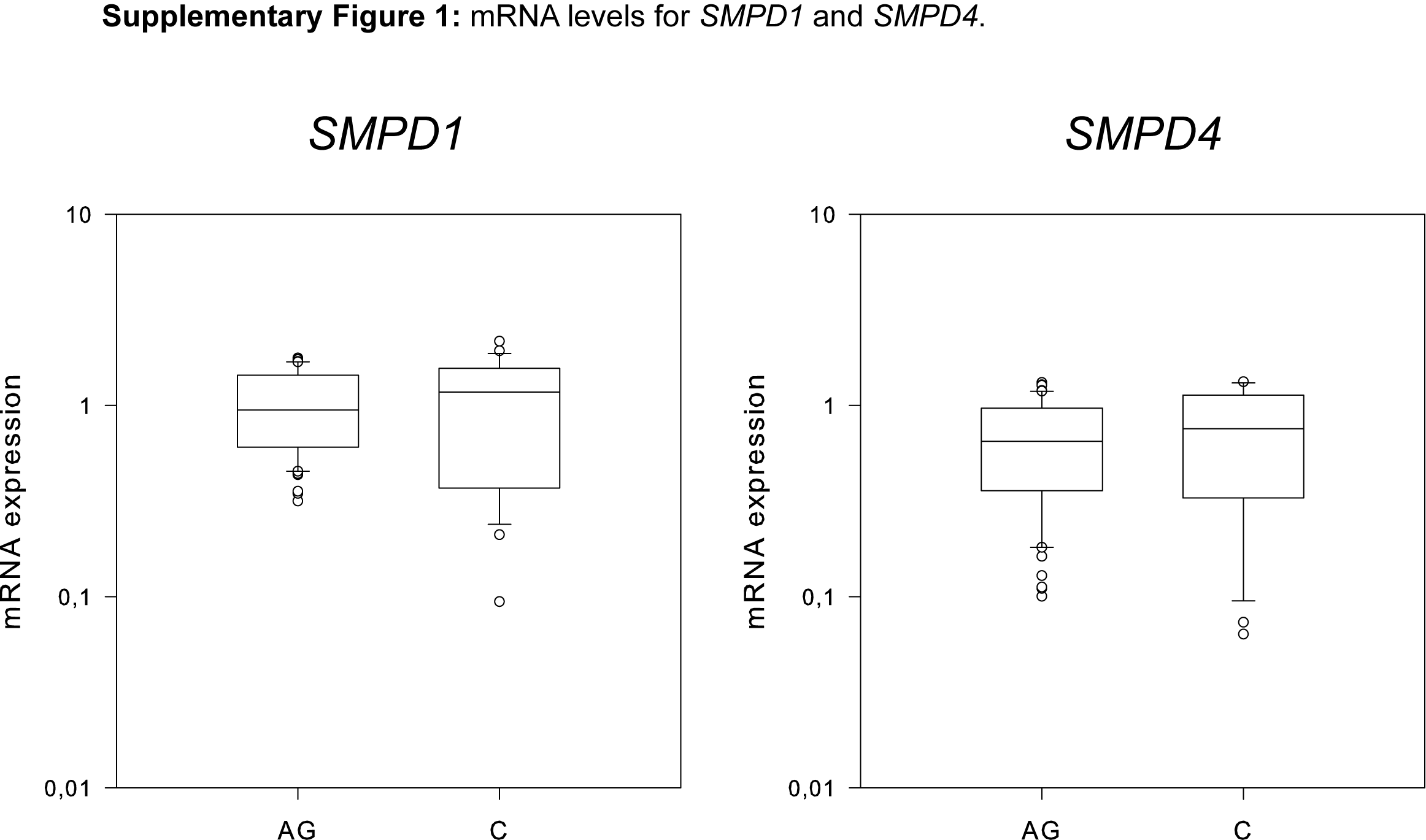

Supplement: Supplementary file 1 [file ijms-23-02428-s001.zip › Supplementary Figure 1.tif]

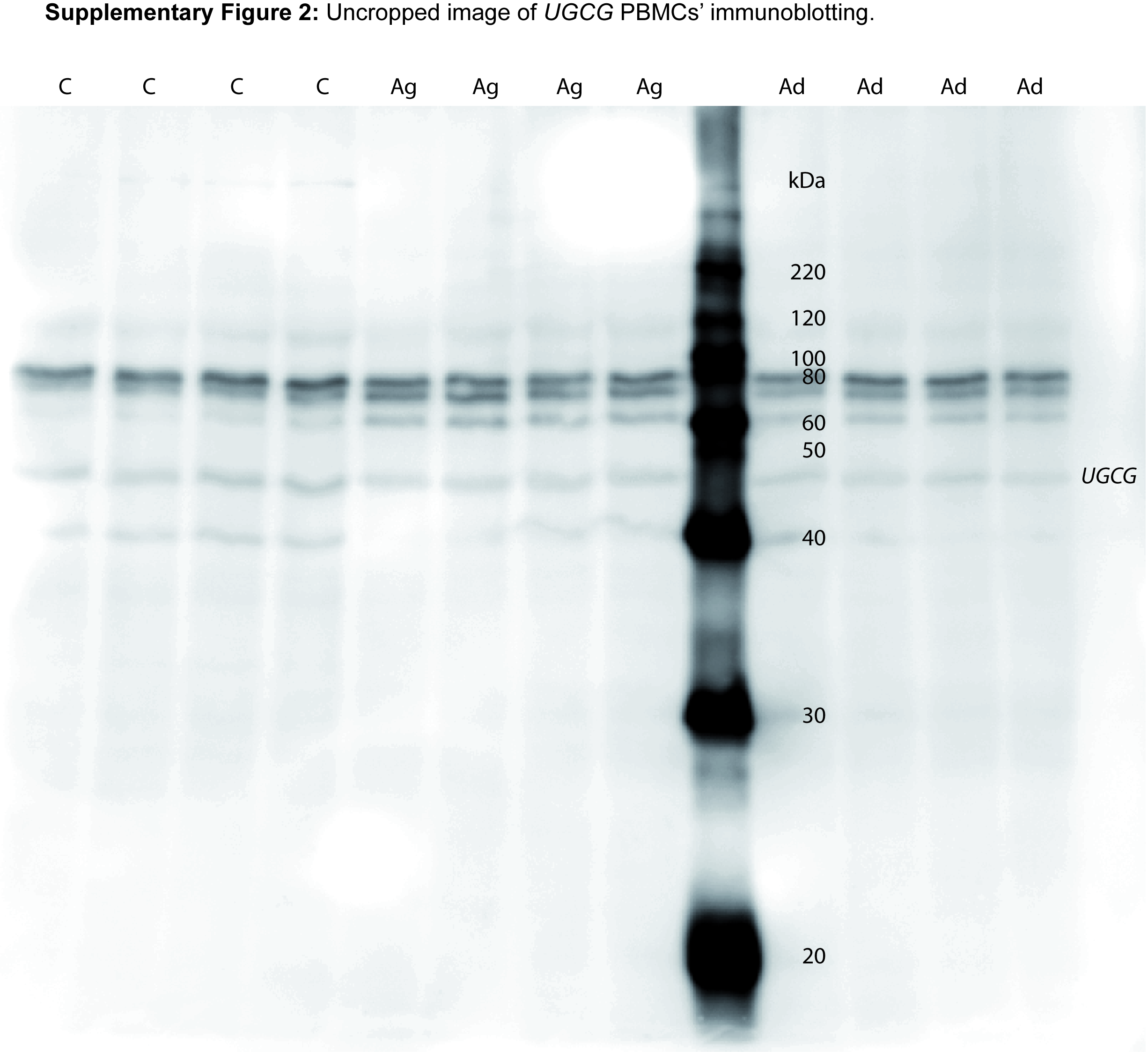

Supplement: Supplementary file 1 [file ijms-23-02428-s001.zip › Supplementary Figure 2.tif]

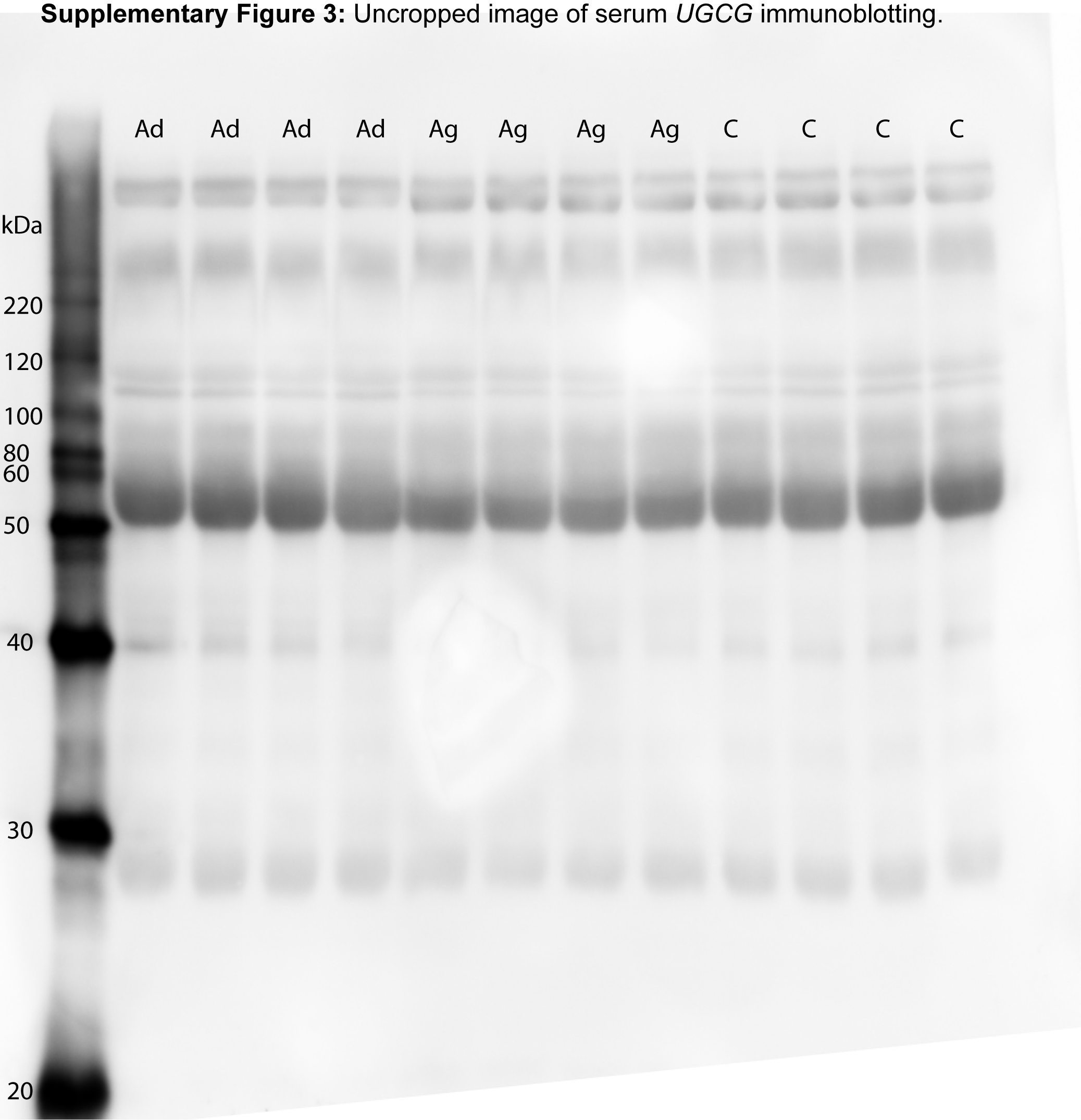

Supplement: Supplementary file 1 [file ijms-23-02428-s001.zip › Supplementary Figure 3.tif]

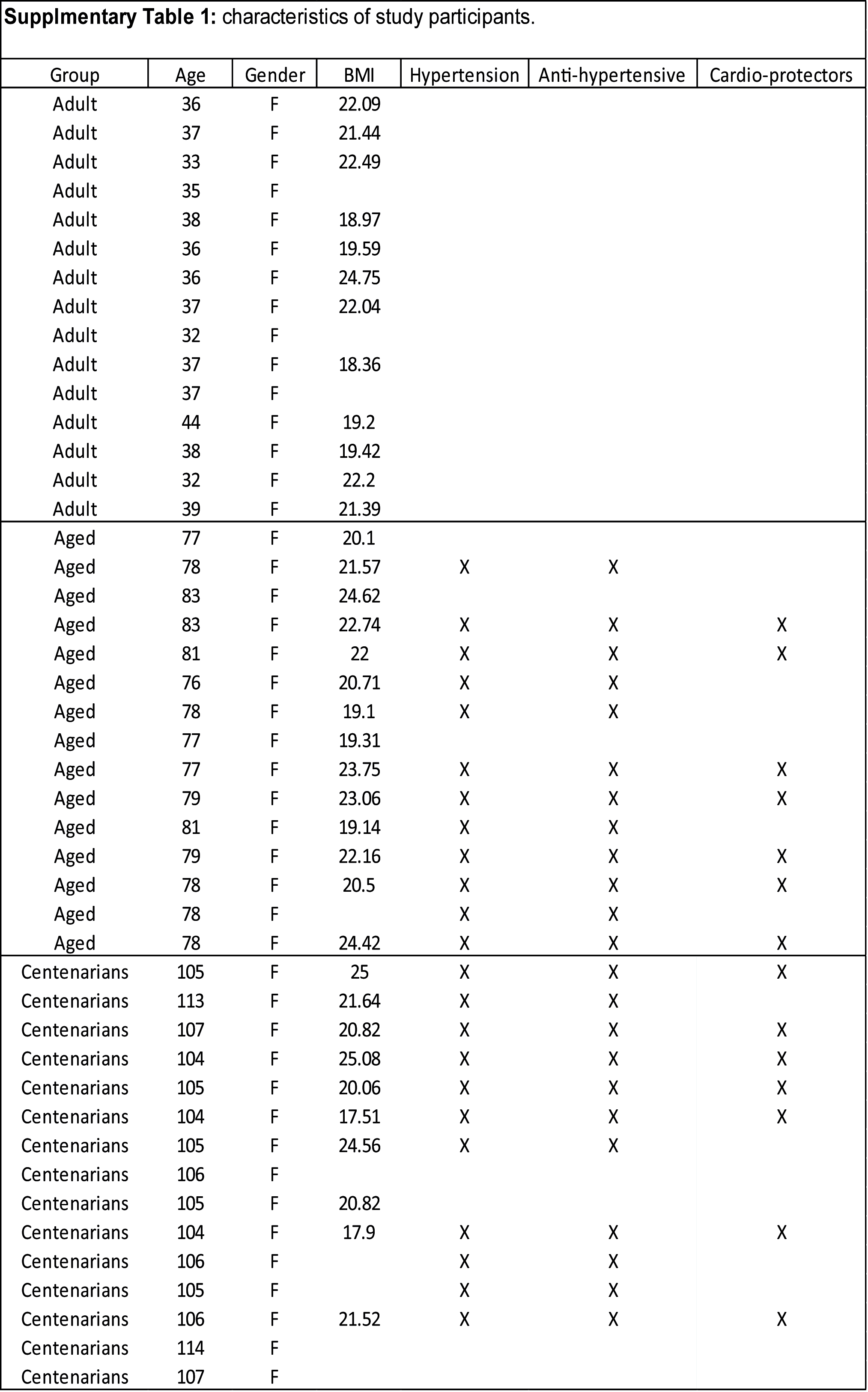

Supplement: Supplementary file 1 [file ijms-23-02428-s001.zip › Supplementary Table 1.tif]

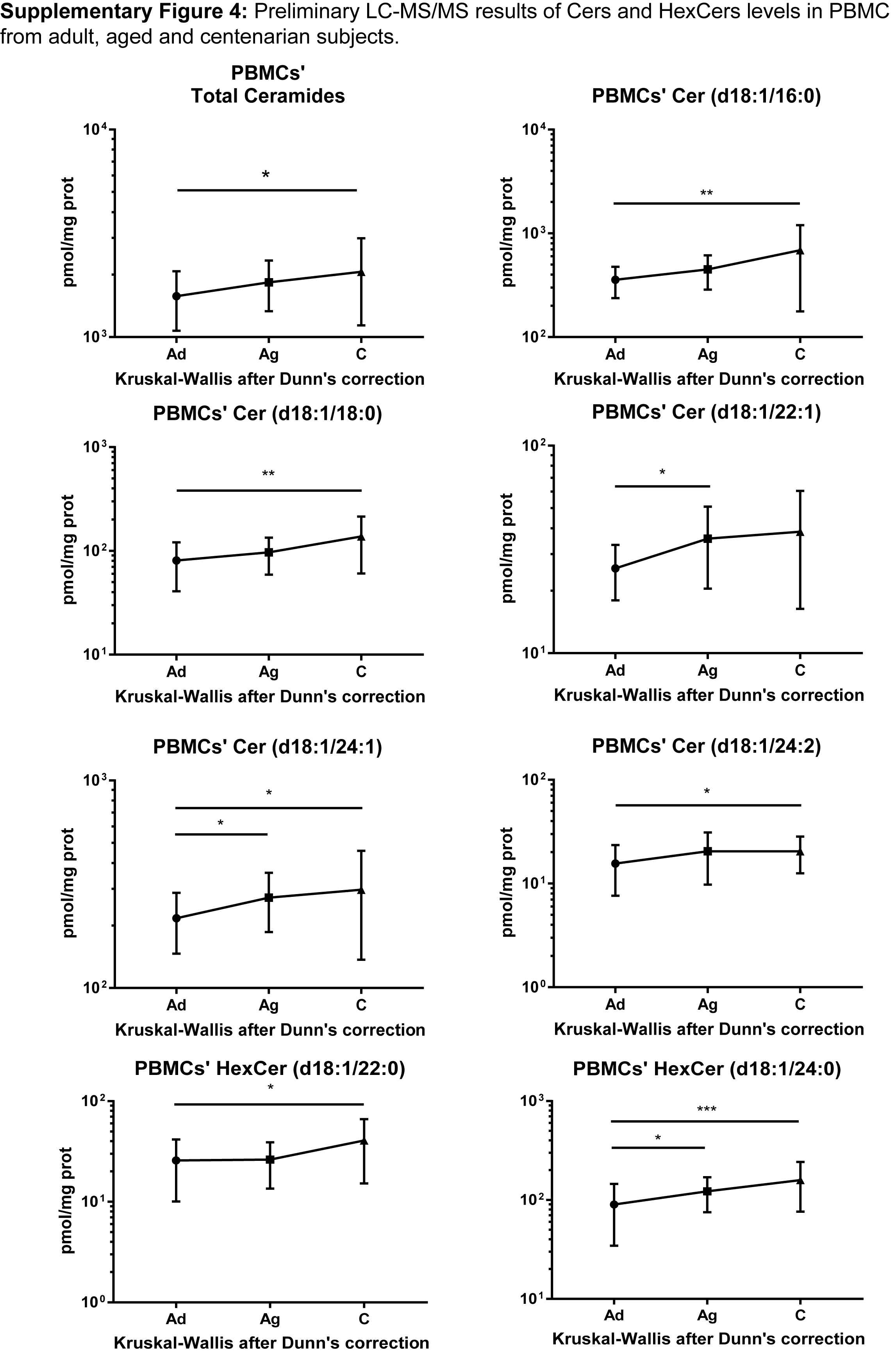

Supplement: Supplementary file 1 [file ijms-23-02428-s001.zip › Supplementary Figure 4.tif]
